# Targeting Bcl2 in cancer

**DOI:** 10.18632/oncoscience.232

**Published:** 2015-09-02

**Authors:** Guo Chen, Xingming Deng

**Affiliations:** Division of Cancer Biology, Department of Radiation Oncology, Emory University School of Medicine and Winship Cancer Institute of Emory University, Atlanta, GA, USA

**Keywords:** Bcl2, BH4, BH3, cancer, therapy

The decision phase of apoptosis is mainly regulated by the Bcl-2 family, which renders these proteins potential targets for cancer therapy through apoptotic mechanisms. Bcl2 family members have homology clustered within four conserved Bcl2 homology (BH) domains (BH1, BH2, BH3 and BH4). Only the antiapoptotic proteins, such as Bcl2, Bcl-XL, Bcl-w and A1, bear the NH_2_-terminal BH4 domain [1]. Mcl-1 does not have a typical BH4 domain but has a helical BH4-like domain which is located between the PEST region and the BH3 domain. The BH1, BH2 and BH3 domains form the surface binding pocket of Bcl2 which mediates protein-protein interactions involving Bcl2 family members. Mutations in this hydrophobic surface binding pocket abolish the antiapoptotic function of Bcl2, demonstrating its requirement in the biological function of Bcl2. To interfere with Bcl2 function, it is necessary to impede binding to the hydrophobic cleft. Synthetic peptides that bind this surface pocket of Bcl-XL and Bcl2 have been shown to induce apoptosis *in vitro*. The hydrocarbon-stapled BH3 peptide not only induces apoptosis but also possesses potent antitumor activity *in vivo*.

Recently, an approach called “structure-activity relationship by nuclear magnetic resonance (SAR by NMR)” has been used for the discovery of novel Bcl2/Bcl-XL inhibitors [2]. In this approach, the relatively large site to be targeted is divided into two smaller half-sites that are individually targeted by small molecules. The two lead molecules are then chemically linked to improve the binding affinity. Subsequent iterative chemical manipulations to improve affinity and decrease binding to human serum albumin, guided by NMR and three-dimensional structures of antiapoptotic proteins, yielded the small molecules ABT-737 and ABT-263, which bind to the hydrophobic pocket of Bcl2 or Bcl-XL with high-affinity and subsequently disrupt the antiapoptotic function of Bcl2 and Bcl-XL with potent anti-tumor effect [2]. Thus, such small molecules that directly target Bcl2 and/or the Bcl2-like proteins by mimicking the BH3 domain should be highly effective anticancer drugs. However, ABT-737 and ABT-263 can induce thrombocytopenia due to their inhibitory effects on both Bcl2 and Bcl-XL [3]. To generate a more selective Bcl2 inhibitor, a tethered indole was incorporated into ABT-263 to fill the P4 hot spot, which specifically interacts with aspartic acid (Asp 103) of Bcl2 but not Glu96 of Bcl-XL, leading to the generation of the Bcl2-selective inhibitor ABT-199 [4]. Since ABT-199 did not cause thrombocytopenia *in vivo* [4], this suggests that selective inhibition of Bcl2 may benefit the development of improved Bcl2 antagonists.

In addition to the hydrophobic surface binding pocket, the NH_2_-terminal BH4 domain (aa-6-31) of Bcl2 is also required for its antiapoptotic function [5]. The BH4 domain of Bcl2 can interact with multiple molecules, including Bax, CED-4, Ras, PP2A, PP2B, IP3 receptor (IP3R), and others. Since only the prosurvival Bcl2 family members possess a conserved N-terminal region denoted BH4, this suggests a critical role of this amphipathic helix for their survival activity. Intriguingly, either caspase-mediated cleavage or mutagenic removal of the BH4 domain not only completely abolishes the antiapoptotic activity of Bcl2 but also results in a conversion of Bcl-2 to a Bax-like death effector [6]. The BH4 domain peptide has been reported to exert antiapoptotic activity *in vivo*, which provides direct evidence that the BH4 domain contributes to the survival function of the prosurvival Bcl2 family members. Since the BH4 domain is critical for the antiapoptotic function of Bcl2, this amphipathic helix should also be an ideal structural target for the screening of small molecules that may bind to this domain and interfere with Bcl2's survival activity. Solution structure of the BH4 domain exhibits multiple potential binding pockets for small molecule docking [7]. Recently, we chose the BH4 domain of Bcl2 as the docking site for screening of small molecules and identified BDA-366 as a Bcl2 BH4 antagonist that is distinct from previous BH3 mimetics. BDA-366 selectively targets the BH4 domain of Bcl2 and converts Bcl2 from a survival molecule to a cell death inducer through a conformational change (BH3 exposure) (Figure 1). BDA-366 not only induces apoptosis but also autophagic cell death of cancer cells by disruption of Bcl2 activity. BDA-366 demonstrates potent antitumor activity in lung cancer xenografts derived from either a lung cancer cell line or a patient-derived small cell lung cancer tumor [8].

In summary, BH3 mimetics (ABT-263 and ABT-199) and the BH4 antagonist (BDA-366) are two different classes of Bcl2 inhibitors that target Bcl2 at the hydrophobic binding pocket or BH4 domain, respectively. Disruption of Bcl2's antiapoptotic function via BH3 mimetics or the BH4 antagonist may represent attractive strategies for cancer treatment.

**Figure 1:**
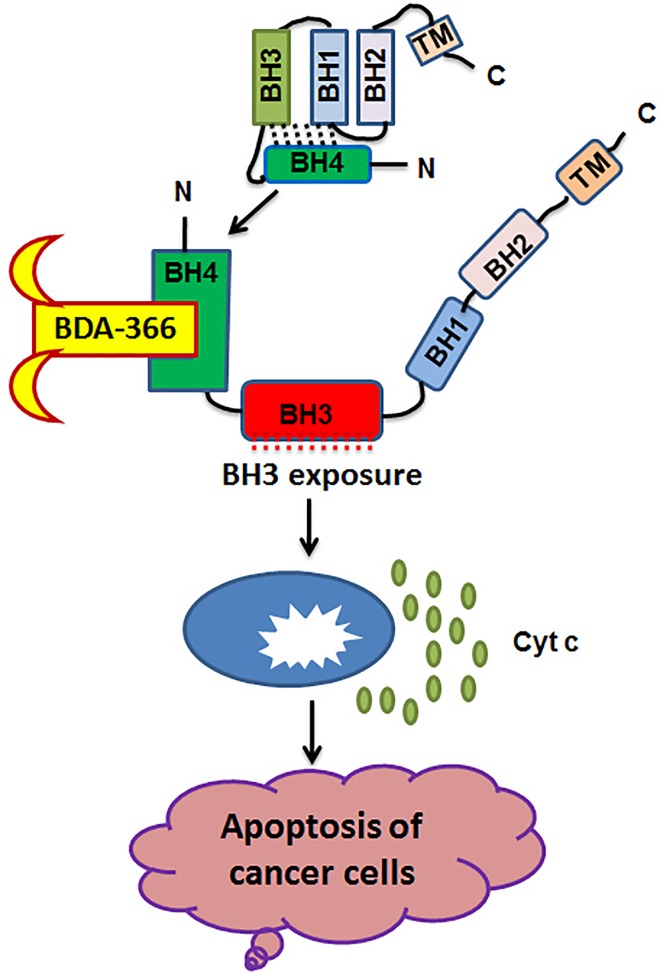
Proposed model of Bcl2 BH4 antagonist BDA-366 induction of apoptosis in cancer cells

## References

[1] Kelekar A (1998). Trends in cell biology.

[2] Oltersdorf T (2005). Nature.

[3] Schoenwaelder SM (2011). Blood.

[4] Souers AJ (2013). Nat Med.

[5] Huang DC (1998). The EMBO journal.

[6] Cheng EH (1997). Science.

[7] Petros AM (2001). Proc Natl Acad Sci U S A.

[8] Han B (2015). Cancer Cell.

